# A biocompatible sol–gel derived titania coating for medical implants with antibacterial modification by copper integration

**DOI:** 10.1186/s13568-018-0554-y

**Published:** 2018-02-19

**Authors:** Hans Gollwitzer, Maximilian Haenle, Wolfram Mittelmeier, Frank Heidenau, Norbert Harrasser

**Affiliations:** 1 Clinic of Orthopedics and Sports Orthopedics, Technische Universität München, Ismaninger Str. 22, 81675 Munich, Germany; 2 ATOS Klinik München, Effnerstr. 38, 81925 Munich, Germany; 3 Klinik für Sportorthopädie und arthroskopische Chirurgie, Orthopädische Fachkliniken der Hessing-Stiftung, Hessingstr. 17, 86199 Augsburg, Germany; 4 Department for Orthopaedic Surgery, University Medicine Rostock, Doberaner Str. 142, 18057 Rostock, Germany; 5 BioCer Entwicklungs GmbH, Birkenstr. 14, 95488 Eckersdorf, Germany

**Keywords:** Sol–gel techniques, Titanium oxide, Metal ion release, Metal ion toxicity, Infection

## Abstract

Implant-associated infections are dangerous complications and may cause dramatic illness with hematogeneous spread of bacteria and secondary infections. Since treatment of these infections remains most challenging and commonly requires implant removal, prevention is of utmost importance. In the present work a titania-sol was equipped with a copper salt resulting after calcination in a titania coating (TiO_2_) with antibacterial properties combined with good cytocompatibility. In vitro tests with bacteria as well as tissue cells were carried out under corresponding conditions. Mouse fibroblasts and different staphylococcal strains were used for growth inhibition assays with serial dilutions of CuCl_2_. Cultivation on the surface of bare Ti6Al4V, TiO_2_-coated and copper-filled TiO_2_-coated Ti6Al4V samples was performed with both bacteria and tissue cells. Bacterial and cellular proliferation and mitochondrial activity were hereby determined. Coating of Ti6Al4V with pure TiO_2_ significantly improved cytocompatibility compared to the uncoated alloy. In the growth inhibition assays, fibroblasts tolerated higher concentrations of copper ions than did bacteria. Nevertheless, copper integration reduced fibroblast proliferation and mitochondrial activity on the surface coating. On the other hand, integration of copper into the TiO_2_-coating significantly reduced adhesion of viable bacteria resulting in a promising combination of cytocompatibility and antibacterial properties. Additionally, significant bacterial growth inhibition by antibacterial amounts of copper was also demonstrated in the supernatant. In conclusion, the copper-loaded TiO_2_-coatings for medical implants may be a promising approach to reduce the rate of implant-associated infections.

## Introduction

Indwelling medical devices such as orthopedic implants have become a growing and indispensable part of modern medicine. Endoprostheses in particular have helped to improve the quality of life of numerous patients. Beside failure due to aseptic loosening and lack of tissue integration, these long-term implants are associated with the problem of bacterial infection by microorganisms frequently introduced during implantation (Horan et al. 1992). Once infected, microorganisms may persist on the implant surface and eradication becomes very critical due to pronounced antibiotic resistance of the adhering bacteria and protection from antibiotic therapy and immune-response by biofilm formation (Gristina 1994; von Eiff and Peters 2003; von Eiff et al. 2002). Despite aseptic conditions during the operation procedure and a peri-operative antibiotic prophylaxis reported infection rates lie between 0.5 and 2% for total hip arthroplasties (THA) (Pedersen et al. 2010; Geipel and Herrmann 2004; Harris and Sledge 1990) and around 1% after total knee arthroplasties (Jamsen et al. 2010; Stefansdottir et al. 2009; Jamsen et al. 2009). Furthermore, infection rates could not be decreased for more than 20 years (Del Pozo and Patel 2009). Thus, novel strategies with effective perioperative prevention of implant colonization by microorganisms are strongly required (Nasser 1994). Multiple surface modifications have been studied to prevent bacterial growth on implants. Anti-adhesive surfaces show promising results on intravascular and intrauretral devices (Pascual 2002; Pechey et al. 2009) with reduction of both bacterial cell and protein adhesion. In orthopedic and trauma surgery however, protein and cellular adhesion are essential for tissue integration and consequently the clinical outcome. Local application of antibiotic drugs is often not only limited by the lack of susceptibility but also an emerging resistance of microorganisms against antibiotics and their clinical significance in orthopaedic surgery (Hidron et al. 2008; Ip et al. 2005; Martinez-Pastor et al. 2010; Haenle et al. 2010, 2011b). These findings emphasize the need of alternative strategies to prevent bacterial implant-associated infections.

Previous studies have shown the bactericidal effect of a titanium dioxide coating loaded with copper ions (Heidenau et al. 2005; Haenle et al. 2011a). Furthermore, its mechanical durability has been demonstrated (Haenle et al. 2011a). The purpose of the present study was to compare cytotoxicity and antibacterial activity of copper ions under corresponding conditions. Furthermore, colonization of the antibacterial TiO_2_-coating for medical implants by tissue cells and bacteria was examined in comparative studies to determine both cytocompatibility and antibacterial properties.

## Materials and methods

### Preparation and characterization of the copper modified TiO_2_-coatings

Commercially available round metal plates of Ti6Al4V alloy (diameter 14.5 mm, thickness 1.5 mm, Goodfellow GmbH, Nauheim, Germany) were used for the colonization studies, silica glass plates were used for scanning electron microscopy (SEM) and energy dispersive X-ray (EDX) analysis to detect titanium and copper. The titanium-oxide coatings were applied in a dip-coating procedure as previously described (Haenle et al. 2011a; Heidenau et al. 2001a, b, 2005). Prior to the coating procedure, the specimens were washed, sonicated in dry ethanol for 2 min and dried with cyclohexane and acetone. Copper ions were incorporated into the sol by cold saturation with copper-(II)-acetate monohydrate (Merck, Darmstadt, Germany). The samples were coated by the dip coating procedure with a speed of 1.5 mm/s and an immersion time of 20 s in the sol. Hydrolysis of the sol combined with film formation was moisture initiated in a controlled atmosphere (40% humidity, 25 °C). After drying the sol film at room temperature for 1 h, calcination was performed in a preheated furnace at 500 °C in air. Multilayer-coated samples may be produced via repetitive dip coating procedures (Haenle et al. 2011a).

SEM-characterization of coated and bare specimens was performed using a Jeol 6400 microscope (Jeol Germany GmbH, Eching, Germany), equipped with a LaB_6_-cathode and a H_igh_P_erformance_G_ermanium_-EDX detector (Explorer, Noran Instruments, Middleton, USA). The data for the layer thickness and the roughness were detected by profilometry (DekTak 3030ST, Veeco, Unterschleissheim, Germany).

### In vitro testing

Initially, the specimens were cleaned in an ultrasonic bath with (1) distilled water, (2) sodium lauryl sulphate solution (2.5% v/v SDS, Life Technologies, Karlsruhe, Germany), (3) extran solution (5% v/v; Sigma, Munich, Germany) and dried with isopropanol. Afterwards, the materials were disinfected under UV light (590 nm) for 2 h (Haenle et al. 2011a; Heidenau et al. 2005).

Microbiological and cell culture studies included the following sample groups:

- uncoated Ti6Al4V plates (Ti6Al4V),
- titanium dioxide coated Ti6Al4V plates (TiO_2_),
- Ti6Al4V plates with a single TiO_2_-coating with integrated copper ions (Cu-TiO_2_) and
- Ti6Al4V plates with a double TiO_2_-coating with integrated copper ions (2×Cu-TiO_2_).

### Special tissue cell preparation

Mouse connective tissue fibroblasts (L929, DSMZ GmbH, Braunschweig, Germany) were used for cell culturing. Cells were precultured with 5% CO_2_ in RPMI 1640 (Gibco Vitrogen Corporation, New York, US) supplemented with 10% (v/v) fetal calf serum (FCS, Life Technologies, Grand Island, US). After 4 h, the culture medium was withdrawn, renewed and the fibroblasts were proceeded to toxicity and biocompatibility testing (Heidenau et al. 2005).

### Special preparation of bacterial strains

Inhibition of bacterial growth by copper ions was evaluated with *S. aureus* ATCC 25923, *S. epidermidis* ATCC 35984 (RP62a) and the strongly biofilm-forming *S. epidermidis* strain ATCC 35983 (RP12). A clinical isolate of a methicillin-resistant *S. aureus* (MRSA 27065) recovered from a patient with an infected total hip arthroplasty and an isolate from an infected central venous line [*S. epidermidis* SE 183 (Gollwitzer et al. 2003)] were identified by standard microbiological techniques and also applied in the growth inhibition assays. The bacterial colonization studies on uncoated and coated Ti6Al4V samples were performed with *S. aureus* ATCC 25923. Biofilm formation was demonstrated by qualitative assessment with the tube assay previously described by Christensen et al. (1985).

Test strains were cultured in trypticase soy broth (TSB, Difco Laboratories, Sparks, US) at 37 °C for 18 h before testing. Bacterial cells were then harvested by centrifugation, washed twice in Dulbecco’s phosphate buffered saline solution, resuspended in normal saline and adjusted by densitometry with a MacFarland 0.5 standard. RPMI 1640 containing 10% (v/v) FCS was infected with the test strain. The final growth medium contained an inoculum concentration of 1.0 × 10^5^ cfu/ml (colony forming units). Aliquots of the final suspension were plated at various concentrations on Mueller–Hinton agar plates for a control of the inoculum counts.

### Growth inhibition tests with copper-(II)-chloride

Growth inhibition tests were performed both for suspended tissue cells and bacterial strains with serial concentrations of CuCl_2_·2H_2_O in the culture medium as previously described (Heidenau et al. 2005). Comparing growth inhibition assays on mouse fibroblasts (L929) and bacteria (*S. epidermidis* ATCC 35984, ATCC 35983, SE 183, *S. aureus* ATCC 25923 and MRSA 27065) were performed under the same conditions to guarantee the same concentrations of free metal ions in the growth medium. Both fibroblast and bacteria concentrations were adjusted to 1.0 × 10^5^ cells/ml and incubated under static conditions at 37 °C in darkness. 24-well polystyrene tissue culture plates (Techno Plastic Products, Trasadingen, Switzerland) were used for all assays.

### Growth inhibition tests with copper-(II)-chloride and tissue cells

Cell proliferation under serial dilutions of CuCl_2_·2H_2_O was determined by quantification of the cells adhering to the culture plates after 24 and 48 h. Attached cells were trypsinized with 300 µl of an aqueous solution containing 0.25% (v/v) trypsin and 0.5 mM EDTA (Sigma, Munich, Germany). The enzymatic reaction was stopped with 700 µl of RPMI 1640 with FCS (10%) and the number of cells was measured with a cell-counter (Coulter Z2, Beckman, Krefeld, Germany).

Cell mitochondric activity as a marker for cell vitality was investigated with the WST-1 test assay (Roche, Basel, Switzerland), measuring the reduction of a tetrazolium salt to formazan. In detail, tissue culture plates with the attached fibroblasts were washed with 1 ml of Dulbecco’s phosphate buffered saline by careful rinsing to remove non-adherent cells followed by incubation for 75 min in a mixture of 750 µl RPMI 1640 with 10% FCS and 10 µl WST-1 ‘2-(4-iodophenyl)-3-(4-nitro-phenyl)-5-(2,4-di-sulfo-phenyl)-2*H*-tetrazolium, monosodium salt’. After complete dissolution of the accumulated formazan in the culture medium, the amount of formazan was quantified with an UV–Vis spectrometer [λ = 430 nm, 690 nm (background), DU 640, Beckman, Krefeld, Germany].

### Growth inhibition tests with copper-(II)-chloride and bacteria

Serial dilutions of CuCl_2_·2H_2_O were also added to the bacterial suspensions and incubated for 24 h. To inhibit reminiscent copper toxicity on bacteria, a neutralizing solution (1.0 g sodium thioglycolate, 1.46 g sodium thiosulfate, 1000 ml deionized water), as described by Tilton and Rosenberg (Heidenau et al. 2005; Tilton and Rosenberg 1978), was added to the growth medium of all samples after the incubation period. Serial dilutions of each sample were plated on Mueller–Hinton agar plates for quantification of viable organisms. Agar plates were incubated for 48 h at 37 °C and the cfu were counted visually.

### In vitro tests with tissue cells in direct surface contact

1 × 10^5^ cells of mouse fibroblasts L929 were inoculated in 1 ml culture medium and incubated for 24 h. The cell number was measured with a cell-counter as described above after detaching the fibroblasts from the surfaces of the samples (Ti6Al4V, TiO_2_, Cu-TiO_2_, 2×CuTiO_2_). Polystyrene wells served as positive control. Similarly, WST-1 test assays were performed to determine mitochondric activity of the fibroblasts adhering to the specimen surfaces.

### In vitro tests with bacteria in direct surface contact

Bacterial adherence to the different surfaces was studied with an assay according to Christensen et al. (1995) with minor modifications (Heidenau et al. 2005). In brief, coated and bare metal plates were immersed in 1 ml of the bacterial suspension with 1 × 10^5^ cfu of *S. aureus* ATCC 25923 and incubated for 24 h. Then, incubation fluid of all samples was removed and supplemented with 1 ml of the neutralizing solution to prevent reminiscent toxicity. Serial dilutions of the incubation fluid were plated on Mueller–Hinton agar plates and incubated at 37 °C for quantification of viable organisms. Cfu were counted visually after 48 h to evaluate antibacterial toxicity of released metal ions in the supernatant growth medium.

After careful rinsing of the colonized metal specimens to remove excessive bacteria, the specimens were placed in vials containing 10 ml of normal saline solution. Sonication for 7 min (Sonorex RK255H, Bandelin Electronic, Berlin, Germany) removed the adhering microorganisms. Serial dilutions of each sample were plated on Mueller–Hinton agar plates and quantified after incubation for 48 h. Complete detachment of the adhering microorganisms was verified through SEM.

### Calculations and statistical methods

Data from bacterial and tissue cell studies were evaluated for statistical significance using non-parametric methods and the method of closed testing procedure (Marcus et al. 1976), with “*P* < 0.05” considered significant (Kruskal–Wallis and Mann–Whitney test).

## Results

### Material characteristics of the copper containing TiO_2_-coatings

**Fig. 1:**
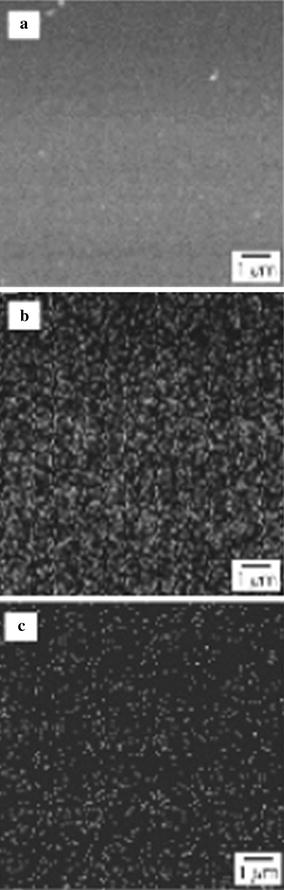
**a** SEM-image of a glass substrate coated with copper filled TiO_2_ (Cu-TiO_2_). **b** Distribution of the Ti_Kα1_ signal of the copper-filled TiO_2_-coating resulting from SEM/EDX investigation. **c** Distribution of the Cu_Kα_ signal of the copper-filled TiO_2_-coating

SEM revealed crack free TiO_2_ layers of pure anatase. A monolayer had a medium thickness of 100 nm, thicker coatings could be achieved by a repetitive coating procedure. Due to the dip coating technique, even irregular and complex surface structures can be coated uniformly. The roughness of the coating was in the range of 3–5 nm (R_a_), reflecting the roughness of the very smooth glass substrate. On the Ti6Al4V material with a roughness of about 0.3 µm (R_a_), the coating showed a smoothing effect in the electron microscopical image, but the effect was not measurable by profilometry. SEM and EDX investigations were carried out on glass substrates to eliminate the influence of the Ti_Kα1_-signal from the Ti6Al4V-alloy. Figure 1a shows an SEM image of the copper-filled TiO_2_-coating (Cu-TiO_2_). Figure 1b, c display the same section but mapped by EDX detection for the single atom species (copper, titanium). Very strong signals from the titanium of the TiO_2_-coating were clearly visible. The copper signals were weaker due to the lower content of the scattering copper species but showed a very uniform distribution within the coating.

### Growth inhibition tests with copper-(II)-chloride in culture medium

**Fig. 2:**
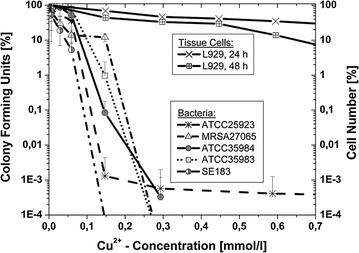
Cell number of fibroblasts L929 and colony forming units of different bacterial strains in dependence of Cu^2+^-content in the culture medium

Growth inhibition diagrams of fibroblasts L929 and the different bacterial strains are visualized in Fig. 2. Growth of bacteria was exponentially inhibited with increasing Cu^2+^-concentrations. Growth curves of all studied staphylococcal strains showed similar characteristics and no strain proved resistant against Cu^2+^ ions. A significant reduction of growth was observed between concentrations of approximately 0.1 and 0.3 mmol/l, and viable counts dropped by an average of 10^5^ cfu/ml. Only single cfu demonstrated tolerance in the presence of high Cu^2+^-concentrations.

**Fig. 3:**
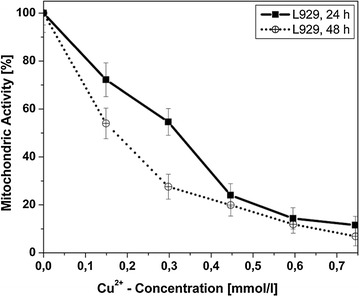
Cell vitality of fibroblasts L929 in dependence of the Cu^2+^-concentration and the cultivation time in the culture medium

Cell counts of L929 decreased with rising concentration of Cu^2+^ ions in the growth medium. Mitochondric activity of the incubated fibroblasts is shown in Fig. 3 and corresponded very well to the cell proliferation. However, even at very high Cu^2+^-concentration of 0.7 mmol/l, more than 10% of the L929 cells remained viable.

### In vitro tests in direct surface contact

To compare the results of the microbiological tests of the different coatings to the values of a clinically established material, all diagrams were standardized to TiAl6V4, which was set 100%.

### In vitro tests with tissue cells in direct surface contact

**Fig. 4:**
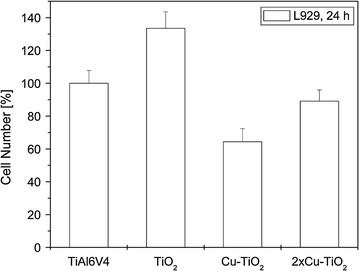
Cell number of fibroblasts L929 on uncoated and the different TiO_2_-coated Ti6Al4V samples

**Fig. 5:**
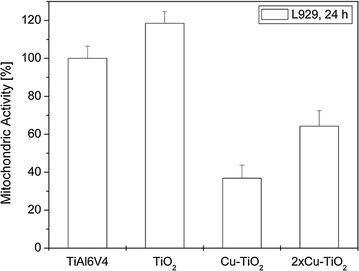
Cell vitality of fibroblasts L929 on uncoated and the different TiO_2_-coated Ti6Al4V samples

Coating of the Ti6Al4V specimens with TiO_2_ significantly increased both the fibroblast growth on the sample surfaces (*P* = 0.01) and the fibroblast mitochondric activity (*P* = 0.02) (Figs. 4, 5).

Integration of copper into the TiO_2_-coating significantly reduced the cell growth (*P* = 0.0007) as well as the cell vitality (*P* = 0.0001) compared to the pure TiO_2_-coating. Coatings with two layers of Cu-TiO_2_ demonstrated significantly higher cell numbers on the sample surface (*P* = 0.015), and higher mitochondric activity (*P* = 0.01) of the adhering cells were found in comparison with the single coated plates.

### In vitro tests with bacteria in direct surface contact

**Fig. 6:**
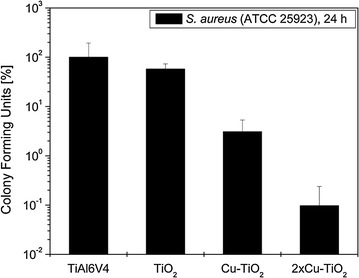
Growth of *S. aureus* ATCC 25923 on uncoated and the different TiO_2_-coated Ti6Al4V

Coating of Ti6Al4V with TiO_2_ alone did not influence bacterial adhesion in vitro after 24 h (Fig. 6). However, a significant reduction of viable adhering staphylococci could be observed on samples coated with a single Cu-TiO_2_ layer (*P* = 0.002). This reduction was even more pronounced with samples coated by two layers of Cu-TiO_2_, reducing viable staphylococci on the surface by the factor 10^3^ compared to uncoated plates. This additional inhibition of bacterial growth was also highly significant compared to the Cu-TiO_2_ monolayer (*P* = 0.002).

### Impact of released ions on bacteria in the supernatant

**Fig. 7:**
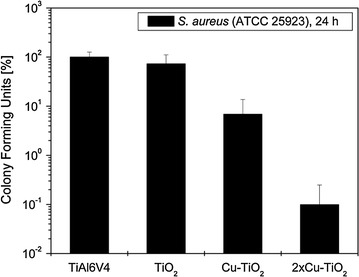
Growth of *S. aureus* ATCC 25923 in the supernatant growth medium for cultivation of uncoated and the different TiO_2_-coated TiAl6V4 samples after 24 h

The impact of the TiO_2_-coatings on planktonic bacteria in the supernatant growth medium was also studied by quantification of viable organisms. After 24 h, a significant reduction of bacterial growth could be observed in the growth medium containing samples with Cu-TiO_2_ surface coatings (*P* = 0.002) (Fig. 7). Reduction was most pronounced with 2×Cu-TiO_2_ and again resulted in an inhibition of staphylococcal growth by the factor 10^3^ (*P* = 0.002).

## Discussion

Antibacterial properties of different metal ions have been studied extensively (Domek et al. 1984; Hassen et al. 2001; Heidenau et al. 2005; Landeen et al. 1989; Morrier et al. 1998; Robertson and Chen 1984; Borkow and Gabbay 2005) and are mainly dependent on the free metal ion concentration. However, cytotoxicity of the applied metal is also strongly related to the free ion concentration (Ramamoorthy and Kushner 1975; Gellert et al. 1999). Therefore, any constituent in the medium (proteins, amino acids, salts, buffers, etc.) and any test parameter (pH value, incubation time, temperature, etc.) influencing the free metal ion concentration may influence the in vitro results (Ramamoorthy and Kushner 1975; Gellert et al. 1999). Furthermore, loss of free metal ions by binding to vial walls has been reported (Landeen et al. 1989). Other differences in toxicity levels and antibacterial properties can be explained by the use of different bacterial strains and tissue cells. The antibacterial capacity of copper ions for example has been intensively studied (Domek et al. 1984; Gellert et al. 1999; Hassen et al. 2001; Landeen et al. 1989; Ramamoorthy and Kushner 1975). However, growth inhibition doses (EC_50_) have enormous variations due to different test parameters with values ranging from 0.007 to 53 mg/l (Gellert et al. 1999).

Corresponding to the microbiological data, various cytotoxicity studies with tissue cells have been published (Puleo and Huh 1995; Schmalz et al. 1998; Yamamoto et al. 1998). For example, Yamamoto et al. evaluated cytotoxicity of 43 metal salts using fibroblasts and osteoblastic cells under standardized conditions to rate the toxic potential of the salts (Yamamoto et al. 1998). In a previous study comparing both activity against bacteria and toxicity against tissue cells under the same and standardized conditions, we were able to demonstrate that copper ions exhibit a most favorable ratio between bactericidal activity and cell toxicity (Heidenau et al. 2005). The present results of the growth inhibition assays clearly demonstrate, that copper ions exhibited a strong activity against planktonic staphylococci at concentrations of 0.1–0.3 mmol/l, with bacterial growth reduced by approximately 5 log_10_. Tissue cell growth and vitality at this concentration were significantly less inhibited. These findings coincide with previous data studying either activity against bacteria or toxicity towards tissue cells (Domek et al. 1984; Gellert et al. 1999; Yamamoto et al. 1998; Heidenau et al. 2005). Nevertheless, a limitation of the present study is the fact, that only mouse fibroblasts rather than human fibroblasts were used for cytotoxicity testing.

Integration of copper ions into the biocompatible TiO_2_ surface coatings significantly reduced adhesion of viable microorganisms on the specimen surfaces and released bactericidal amounts of copper ions to the supernatant growth medium. Obviously, copper ions were also released from the deeper layer of the copper-filled TiO_2_ double layer, enhancing bacterial killing both on the coating surface and in the supernatant growth medium resulting in a further reduction of bacterial growth. This effect has already been described for mulit-layer copper-TiO_2_-coatings and different bacterial strains (Haenle et al. 2011a; Heidenau et al. 2005). This effect might be due to an almost linear increase of copper ions measured within the growth medium (Haenle et al. 2011a). As expected, an antibacterial effect of the pure TiO_2_ could not be observed. This observation is most relevant, since in the clinical leaching of copper ions from the coatings could also reach microorganisms located in the periimplant tissue. This could effectively protect the implant from bacterial colonization during the operation and in the early postoperative period, when the risk for an implant-related infection is highest (Geipel and Herrmann 2004; Nasser 1994). Furthermore, since implants are not vascularized, antibiotics and immune cells reach the implant surface only by diffusion (Cordero and Garcia-Cimbrelo 2000).

In the tissue cell tests, the pure TiO_2_-coating significantly improved both surface cell growth and vitality of fibroblasts L929 on the coated surfaces compared to standard Ti6Al4V specimens. These findings coincide with previous findings where the pure TiO_2_-coating significantly improved cell proliferation of Embryo calvaria mouse osteblast like cells (MC3T3-E1) (Heidenau et al. 2005). However, incorporation of antibacterial amounts of copper reduced tissue cell proliferation and vitality compared to the pure TiO_2,_ but to a minimized extend. Copper loaded double layers even show a cell behavior comparable to that on uncoated metal surfaces. The improved cytocompatibility of the copper-loaded TiO_2_ double layer (2×Cu-TiO_2_) in comparison to the copper-loaded TiO_2_ monolayer (Cu-TiO_2_) may be explained by advantageous surface properties, e.g. an improved surface topography. Due to the essentially slower metabolism and proliferation rate of fibroblasts L929 compared to bacteria, elevated levels of metal ions like copper injure the faster growing bacteria to a much higher degree. After the release of the Cu^2+^ ions, the pure biocompatible TiO_2_ surface coating remains, promoting tissue integration.

The bactericidal effect of metal ions, especially copper and silver ions, is a well known phenomenon which has been recognized for centuries (Dollwet and Sorenson 1985). Pure copper and silver films have to be discussed critically in orthopedic and trauma surgery where tissue integration is essential for permanent implants, because the permanent long-term release of ions may result in reduced tissue integration of the implant and a pronounced “immuno-incompetent fibro-inflammatory zone” (Gristina 1994). Modification of the biocompatible TiO_2_-coating by copper incorporation is hence only one possibility to create antibacterial properties. In this respect, amongst others silver-coated orthopaedic implants have been studied. In animal experiments as well as in a prospective clinical trial a reduced infection rate was thereby observed (Hardes et al. 2010; Gosheger et al. 2004). However, this very silver coating may not be applied to the prosthetic stem, i.e. the anchoring parts of the prosthesis (Hardes et al. 2010). On the other hand, with the presented copper-loaded TiO_2_ surface coating, infection prevention could be achieved in the perioperative period, combined with excellent biocompatibility that allows coating of implant parts made for implant fixation in bone and other tissues. Furthermore, the remaining biocompatible surface coating could improve immuno-competence at the interface between implant and host tissue reducing both the risk for infection and aseptic loosening (Gristina 1994).

In summary, coating of standard titanium alloys with a sol–gel derived titanium dioxide coating (TiO_2_) improved surface colonization by tissue cells. Adhesion of multiple strains of viable staphylococci was reduced significantly by a single copper-loaded TiO_2_-coating. Integration of antibacterial amounts of copper reduced cytocompatibility of the coating only to a minor degree. Best antibacterial properties were reached with a copper-loaded TiO_2_ double layer that showed a cytocompatibility comparable to Ti6Al4V. The antibacterial and biocompatible TiO_2_ surface coating could improve tissue integration and similarly reduce the risk for infection as well as aseptic loosening of hard tissue implants.

## Abbreviations

CFU: colony forming units
Cu-TiO_2_: Ti6Al4V plates with a single TiO_2_-coating with integrated copper ions
2×Cu-TiO_2_: Ti6Al4V plates with a double TiO_2_-coating with integrated copper ions
EDX: energy dispersive X-ray
SEM: scanning electron microscopy
Ti6Al4V: uncoated Ti6Al4V
TiO_2_: titanium dioxide coated Ti6Al4V
